# Multivariate Analysis and Machine Learning in Cerebral Palsy Research

**DOI:** 10.3389/fneur.2017.00715

**Published:** 2017-12-21

**Authors:** Jing Zhang

**Affiliations:** ^1^Department of Neurology, Washington University in St. Louis, St. Louis, MO, United States

**Keywords:** multivariate analysis, machine learning, cerebral palsy, early diagnosis, outcome assessment

## Abstract

Cerebral palsy (CP), a common pediatric movement disorder, causes the most severe physical disability in children. Early diagnosis in high-risk infants is critical for early intervention and possible early recovery. In recent years, multivariate analytic and machine learning (ML) approaches have been increasingly used in CP research. This paper aims to identify such multivariate studies and provide an overview of this relatively young field. Studies reviewed in this paper have demonstrated that multivariate analytic methods are useful in identification of risk factors, detection of CP, movement assessment for CP prediction, and outcome assessment, and ML approaches have made it possible to automatically identify movement impairments in high-risk infants. In addition, outcome predictors for surgical treatments have been identified by multivariate outcome studies. To make the multivariate and ML approaches useful in clinical settings, further research with large samples is needed to verify and improve these multivariate methods in risk factor identification, CP detection, movement assessment, and outcome evaluation or prediction. As multivariate analysis, ML and data processing technologies advance in the era of Big Data of this century, it is expected that multivariate analysis and ML will play a bigger role in improving the diagnosis and treatment of CP to reduce mortality and morbidity rates, and enhance patient care for children with CP.

## Introduction

Cerebral palsy (CP) is the most common movement disorder in children (1) and causes the most severe physical disability in neurodevelopmental disorders (2). Among children with CP, around 33% of them can not walk, 25% can not talk, 25% have epilepsy, 50% have an intellectual disability, and most of them are in pain (3). Spastic CP is the most common subtype of this disorder, often shown as muscle stiffness that causes movement difficulties in a hand, arm, foot, or leg on one or both sides of the body, affecting the majority (>85%) of children with CP (4). Other subtypes of CP include dyskinetic (athetoid or dystonic) CP, ataxic CP (e.g., with tremors), and mixed CP.

Currently, there is no cure for CP, but medications (such as baclofen and botulinum toxin), supportive treatments (such as physical therapy), and surgical procedures [such as orthopedic surgery and selective dorsal rhizotomy (SDR)] can help patients alleviate symptoms and improve motor skills (5). The signs and symptoms of CP usually appear in the early months of life, but the average age for diagnosis of CP is around 2 years (2). Therefore, early identification and intervention is crucial for patients with CP because infants have higher potential for recovery from neural lesions than adults (1, 2). Neuroimaging, motor assessment (such as general movement assessment), and neurological examinations can help identify high-risk infants, monitor neurodevelopment, and detect or predict CP. Neuroimaging such as magnetic resonance imaging (MRI) and cranial ultrasound are useful to detect structural changes [intraventricular hemorrhage, periventricular leukomalacia (PVL), etc.] in the newborn brain, monitor lesion progression, and assess treatment effects, although compared with MRI, cranial ultrasound is less sensitive to lesions in the gray matter or malformations. Severe CP (caused by severe brain lesions such as PVL) can be identified by MRI or cranial ultrasound as soon as the lesions become recognizable on imaging after birth. However, 12–14% of children with CP have negative MRI scans due to subtle lesions in the brain (6). Thus, an integrated approach (imaging, motor assessment and neurological examinations) is needed to predict mild or moderate CP.

To predict CP in infants, Prechtl has described a general movement assessment method as a clinical assessment approach to identify CP motor impairments in infants by evaluating their spontaneous general movements (7). In particular, two atypical motor development features [(1) the presence of cramped-synchronized general movements at a preterm or term age and (2) the absence of small smooth movements or fidgety movements at 3–5 months] have been defined (8–10), which can identify CP in high-risk infants reliably (11). However, only well-trained physicians can perform such assessment, and general movement assessment based on visual observation by physicians is often influenced by subjective impressions and observer fatigue. Therefore, there is growing interest in developing multivariate and machine learning (ML)-based movement assessment tools for a more objective and quantitative motor assessment to detect movement impairments in high-risk infants (12, 13).

Multivariate analysis is a statistical analytical approach that simultaneously evaluates multiple variables, which compared with univariate analysis, may have more advantages (e.g., free from restrictions of various assumptions in univariate analysis) in identifying the associations between multiple data variables (e.g., variables associated with CP outcomes), grouping data into different groups or subgroups (e.g., different CP subtypes), and developing new diagnostic tests (e.g., differentiate CP subtypes with key feature variables). Multivariate analysis includes statistical methods such as principal components analysis (PCA), canonical variate analysis, independent components analysis, and multivariate regression. ML (or statistical learning) is a group of multivariate analytic methods that first identify the most significant data features or patterns that can best separate the data into different classes in the training dataset, and then apply these data features or patterns to the test dataset for data classification or prediction. ML has been increasingly applied to the biomedical field (14, 15), and examples of ML methods include linear discriminate analysis (16), support vector machine (SVM) (17), artificial neural networks (ANN) (18), random forest (19), and cluster analysis (20).

With growing interests, multivariate analysis has been increasingly employed in CP research in recent years, and research with multivariate analyses in CP is in infancy (14). To provide an overview of this relatively young field, PubMed search was performed with keywords “multivariate analysis cerebral palsy pediatric,” “machine learning cerebral palsy,” or “multivariate analysis cerebral palsy imaging.” The search yielded 126 articles. Articles were excluded if their subjects were not pediatric or the statistical methods used were not multivariate or the article was published before year 1990. This paper assessed the studies that used multivariate analysis in CP research and found that multivariate studies in CP are mainly in four categories: (1) risk factor identification; (2) detection of CP and identification of CP abnormalities; (3) movement assessment for CP prediction; and (4) outcome evaluation.

## Multivariate Analysis in Risk Factor Identification

Early work on CP risk factor identification started from birth certificates. In a large population-based cohort study, data from birth certificates for 192 children with CP in four counties in California were compared with 155,636 healthy children in the same regions and the study found that low birth weight and (early or late) gestational age at birth were associated with high prevalence of CP, but early prenatal care and delivery at a hospital (for low birth weight neonates) were not associated prevalence of CP (21). Using multivariate analysis on clinical data of 113 CP infants (identified from 1,105 infants), Pinto-Martin et al. found that in low birth weight infants, cranial ultrasound imaging abnormalities such as parenchymal echodensities/lucencies (or ventricular enlargement) and germinal matrix/intraventricular hemorrhage were strong risk factors for disabling CP, but factors such as birth weight, gestational age, and Apgar score were not associated with it (22). In addition, a multicenter, large sample study (across eight European study centers, *n* = 585) revealed that there was a high rate of infection in mothers of CP children during their pregnancy and major CP abnormalities on structural MRI included white-matter damage due to immaturity (e.g., PVL) (42.5%), lesions in the basal ganglia (12.8%), cortical or subcortical lesions (9.4%), and malformation (9.1%) (23). A number of studies that identified CP risk factors have performed both univariate and multivariate analyses (24–26) where the risk factors identified by multivariate analyses were a subset of those identified by univariate analyses (24, 25), and the results of multivariate analyses were more rigorous and valid.

Studies that applied multivariate analysis to CP risk factor identification are summarized in Table 1 (22, 24–31). Multivariate logistic regression has often been used to identify risk factors for CP (22, 26, 29–31). Some of the risk factors identified by multivariate analytic studies include: premature birth, low birth weight, severe birth asphyxia, preterm rupture of membrane, abnormal cranial ultrasound, or structural MRI imaging findings (e.g., parenchymal echodensities/lucencies, or ventricular enlargement), intraventricular hemorrhage, PVL, neonatal sepsis, hypoxia-ischemic encephalopathy, hypoglycemia, neonatal jaundice, etc. (22–31). These risk factors are useful to understand the causes of CP, identify high-risk infants, and aid in the diagnosis of CP.

**Table 1:** Summary of studies with multivariate analyses in identification of risk factors and detection of CP.

Study	Subject sample	Data	Methods	Main findings	Other findings
Pinto-Martin et al. (22)	113 children with CP	Clinical data (birth weight, gestational age, length of hospital stay, gender, race, plurality, presence of labor, Apgar score, motor function, cranial US findings, etc.)	Multivariate logistic regression to assess risk factors for CP	Risk factors for disabling CP: PEL/VE or ventricular enlargement on cranial US, germinal matrix/intraventricular hemorrhage, mechanical ventilation; risk factors for non-disabling CP: PEL/VE	Cranial US abnormalities are strong risk factors for disabling CP in low birth weight infants; non-risk factors for disabling CP: birth weight, gestational age, length of hospital stay, gender, race, plurality, presence of labor, Apgar score
Allan et al. (24)	36 pts with CP (in 381 infants)	Clinical data (birth weight, bronchopulmonary dysplasia, abnormal cranial US findings, treatment, etc.)	Univariate and multivariate analysis to identify antecedents of CP	Predictors of CP: bronchopulmonary dysplasia and an abnormal cranial US scan (showing grade 3 to 4 intraventricular hemorrhage, PVL, or ventriculomegaly)	PVL and ventriculomegaly associated with high CP detection rates; chorioamnionitis and treatment with surfactant significant in univariate analysis
Kim et al. (25)	35 pts with CP	Clinical data (age, weight, neonatal sepsis, neonatal seizure, etc.)	Univariate and multivariate analysis to identify risk factors for CP	Risk factors for CP and delayed development: neonatal sepsis	
Han et al. (27)	21 children with CP	Clinical data (birth characteristics, disease at birth, neonatal cerebral ultrasound findings, etc.)	Multivariate analysis used to identify risk factors for CP	Risk factors for CP: existence of PVL, preterm labor, preterm rupture of membrane, severe birth asphyxia, neonatal sepsis, and respiratory distress syndrome	Existence of PVL is the strongest risk factor for CP
Zhong et al. (28)	308 children with CP	Data from a cross-sectional survey (birth characteristics, disease during the first month of life, etc.)	Multivariate analysis used to identify risk factors for CP	Risk factors for CP: delivery at home, low Apgar score, illness during the first month of life, maternal cold with fever in early gestation, low protein intake during pregnancy, low education level of mother	
Golomb et al. (26)	76 children with CP after perinatal stroke	Clinical data (perinatal history, motor function, frequency of CP, degree of disability, etc.)	Univariate and multivariate analysis (with logistic regression) to assess risk factors for CP in perinatal stroke	68% pts with perinatal stroke had CP; risk factors for CP: delayed stroke and male gender; In pts with neonatal stroke, risk factors for triplegia or quadriplegia: bilateral infarcts	In pts with unilateral middle cerebral artery infarcts, risk factors for CP: delayed stroke and large-branch infarction
Miamoto et al. (29)	60 pts with CP vs. 60 healthy controls	Data from questionnaires and clinical exams (TMD symptoms, bio-psychosocial characteristics, etc.)	Multivariate logistic regression to determine risk factors for TMD symptoms	Risk factors for TMD symptoms: presence of CP, male gender, severity of the malocclusion, mouth breathing, and mixed dentition	13.3% pts vs. 1.7% controls had TMD symptoms
Abdullahi et al. (30)	111 pts with CP vs. 222 controls	Clinical data (maternal sociodemographic characteristics and neonatal expected predictors)	Univariate and multivariate (logistic regression) analyses used to identify factors associated with CP	Predictors of CP: maternal fever, previous neonatal death, and poor sucking	Factors not associated with CP: maternal age, parity, birth weight, and sex
Yu et al. (31)	203 preterm infants with CP, vs. 220 preterm infants without CP or other neurological disorders	Data of diseases of premature infants, the treatments in neonatal period, etc.	Multivariate logistic analysis used to identify risk factors associated with CP	Risk factors for CP: occurrence of PVL, HIE, hypoglycemia, or neonatal jaundice	Continuous positive airway pressure may lower the risk of CP
Golomb et al. (32)	76 children with CP after perinatal stroke	Clinical data (perinatal history, motor function, frequency of CP, degree of disability, etc.)	Univariate and multivariate analysis (with logistic regression) to assess association of CP with other disabilities	72% pts with perinatal stroke had at least another disability; risk factors for epilepsy: neonatal presentation and history of cesarean-section delivery	Risk factors for severe cognitive impairments or epilepsy: perinatal stroke with neonatal presentation
Griffiths et al. (33)	20 pts with spastic CP; 20 with dyskinetic CP	Injury severity scores at different brain regions on magnetic resonance imaging (T2)	Variables indicated by univariate analysis fed to multivariate logistic regression to identify predictors to differentiate spastic and dyskinetic CP	Spastic CP pts had more severe damage to white matter near the paracentral lobule; dyskinetic CP pts had more injury to the STN: hypoxic-ischemic injury to the STN at birth associated with dyskinetic CP	Non-predictors of dyskinesia: injuries to the putamen, caudate, and globus pallidus
Yoshida et al. (34)	34 pts with CP vs. 21 healthy subjects	Parameters (number of fibers, tract-based FA, and FA) for CST and posterior thalamic radiation tracts from diffusion tensor imaging (DTI) and motor level data	Univariate and multivariate (regression) analysis used to identify variables correlated to gross motor function	Number of fibers and ROI-based FA values of both tracts were lower in pts than controls; motor-sensory parameters were negatively correlated with GMFCS level	
Coppola et al. (35)	Group 1: 40 pts with CP and mental retardation; group 2: 47 pts with CP, mental retardation and epilepsy; group 3: 26 pts with epilepsy	Clinical data (age, BMI, BMD *z*-score from dual-energy X-ray absorptiometry scan, etc.)	Multivariate analysis used to identify factors on BMD	Lower BMD in 42.5% pts in group 1, 70.2% in group 2, 11.5% in group 3	In pts with CP, mental retardation and epilepsy, epilepsy is an aggravating factor on bone health Factors on BMD: age, BMI, severe mental retardation, epilepsy
Benfer et al. (36)	120 pts with CP	Data of OPD measures, motor measures, etc.	Univariate and multivariate regression analysis to determine the relationship between OPD and motor functions	Higher odds of OPD in non-ambulant pts than in ambulant pts	85% pts had OPD
Romeo et al. (37)	100 pts with CP (32 of them with epilepsy) vs. 100 healthy children	Data from the SDSC, GMFCS levels, etc.	Multivariate analysis (logistic regression) used to identify factors associated with SDSC	13% of children with CP had abnormal sleep score; factors associated with SDSC: behavioral problems and epilepsy	Compared with healthy controls, sleep disorders are more common in children with CP
Adler et al. (38)	18 children with unilateral spastic CP (9 with mirror movement, 9 without)	Clinical data from BANIMM, JTHFT, and AHA	Multivariate analysis of covariance used to determine whether mirror movements affect daily living	Mirror movements had a negative impact on bimanual performance (AHA) and on the time needed to complete difficult activities	
Tao et al. (39)	11 children with CP, 8 healthy children, 7 healthy adults	EMG data from five thigh muscles and three lower leg muscles	Multivariate empirical mode decomposition enhanced MMSE analysis used to analyze EMG data; repeated-measure ANOVAs for group comparison	Compared with the control group, CP pts had distinct diversity in MMSE curve	Abnormal MMSE curve reflected problems in individual muscles such as motor control impairments, loss of muscle couplings, and spasticity or paralysis
Ghate et al. (40)	54 pts with CP	Clinical data (CP type, motor function, etc.) and data from ophthalmoscopic examinations	Multivariate logistic regression to identify factors associated with motor outcomes	70% pts had abnormal optic nerve head; disk pallor associated with non-ambulatory status and quadriplegia; large cup associated with age at examination	Indicator for poor motor outcome: presence of optic nerve head pallor
Reid et al. (41)	31 children with unilateral CP	Activation maps from fMRI (with hand task); FA and MD values and fiber tracts in the thalamocortical and corticomotor tracts from DTI; clinical scores of motor ability	k-means clustering used to identify fMRI-task-specific DTI tracks; surface-based approach (using surface-meshes) compared with voxelwise fMRI-DTI approach; correlation analysis between DTI metrics and clinical scores performed	DTI metrics and five clinical scores of motor function were correlated; surface-based approach processed more subjects’ data (87%) than the voxel-based approach (65%), generated more coherent tractography	Surface-based approach revealed more significant correlations between DTI metrics and five clinical scores
Tosun et al. (42)	30 pts with CP only; 54 pts with epilepsy only; 38 pts with CP + epilepsy; 30 healthy children	BMD of lumbar vertebrae obtained by dual energy X-ray absorptiometry; clinical data (dietary Ca intake, whether intellectual disability, whether immobility, etc.)	Multivariate regression analysis used to evaluate the relationship between BMD and possible risk factors	Low BMD common in pts with CP and CP + epilepsy; risk factor of low BMD: immobility (not able to walk independently)	Low BMD related to the severity of CP, but not to vitamin D levels or AED treatment

*AHA, assisting hand assessment; BANIMM, bimanual activities negatively influenced by mirror movements; BMD, bone mineral density; BMI, body mass index; CST, corticospinal tract; CP, cerebral palsy; EMG, electromyographic; FA, fractional anisotropy; GMFCS, Gross Motor Function Classification System; HIE, hypoxia-ischemic encephalopathy; JTHFT, Jebsen taylor hand function test; MD, mean diffusivity; MMSE, multivariate multi-scale entropy; OPD, oropharyngeal dysphagia; PEL/VE, parenchymal echodensities/lucencies; Pts, patients; PVL, periventricular leukomalacia; ROI, region of interest; SDSC, Sleep Disturbance Scale for Children; STN, subthalamic nucleus; surgeon volume, the number of procedures performed; TMD, temporomandibular disorders; US, ultrasound*.

Further, the risk factors for CP revealed by the multivariate studies are useful to prevent CP. Several CP risk factors such as brain injury and infection can be managed and avoided by preventing their causative mechanisms, and preventive efforts such as rubella vaccination, anti-D vaccination, and preventing methylmercury contamination are effective in preventing CP (2). In addition, meta-analysis has indicated that CP may be reduced by 30% in premature infants (<32 weeks gestation) by providing mothers of imminent-labor with magnesium sulfate for neuroprotection of their babies (43, 44). Further, early interventions such as hypothermia have prevented CP in 12.5% of infants with neonatal encephalopathy following an acute intrapartum hypoxic event (45). Since, currently, there is no cure for CP, CP prevention is critical to reduce the prevalence of CP and save children from CP and CP-caused life-long disabilities. Multivariate analysis may help identify significant risk factors and early interventions in order to prevent CP.

Taken together, multivariate analysis is important in identification of risk factors for CP and the risk factors identified such as premature birth and abnormal (cranial ultrasound or structural MRI) imaging findings are useful not only for CP cause identification and diagnosis but also for CP prevention. Further research is needed to identify more manageable and avoidable risk factors and early interventions (such as neuroprotective drugs or therapies) to prevent CP and reduce CP morbidity rate.

## Multivariate Analysis in Detection of CP and Identification of CP Abnormalities

Since the current average age for diagnosis of CP is around 2 years and infants have higher potential for neural recovery (1, 2), early detection of CP is critical to make early intervention possible. To identify CP in high-risk infants, neuroimaging such as cranial ultrasound and MRI is important for lesion detection and deciding the timing of the lesion. Studies applied multivariate analytic methods to detection of CP and identification of CP abnormalities are summarized in the latter part of Table 1 (32–42). A multicenter study of very-low birth weight infants (*n* = 381, survival rate = 87%, 36, or 9.4% with CP) indicated that cranial ultrasound findings such as grade 3–4 intraventricular hemorrhage and PVL were useful in predicting CP; in particular, PVL and ventriculomegaly were related to high detection rate (≥30%) for CP (24). Further, to differentiate CP subtypes, Griffiths et al. examined the T2 MRI images of children with spastic or dyskinetic CP (*n* = 20 in each group), and found that patients with spastic CP had more severe injury to white matter near the paracentral lobule, while patients with dyskinetic CP had more injury to the subthalamic nucleus (STN) (33). Multivariate logistic regression further identified the associated factors (i.e., lesions in distinctly different anatomical locations) for differentiation of spastic and dyskinetic CP (33).

When brain injuries in patients with CP are subtle, advanced imaging such as diffusion tensor imaging (DTI) and diffusion-weighted imaging is useful to detect CP injuries with subtle abnormalities. DTI metrics such as mean diffusion (MD) and fractional anisotropy (FA) are often used to identify injuries in white matter tracts. The value of DTI in identifying degenerative changes in patients with spastic CP due to periventricular white matter injury has been demonstrated by an early study, which reported that children with spastic hemiparetic CP (caused by periventricular white matter injury) had reduced DTI fiber count on the ipsilateral (the same side as the lesion) side of the corticospinal tract (CST), corticobulbar tract (CBT), and superior thalamic radiation, and had MD and FA changes reflected neurodegeneration of the motor and sensory pathways (*n* = 5) (46). Further DTI studies found that white matter damage in the posterior thalamic radiation pathways was more severe than that in the CSTs in children with CP (*n* = 28) (47), and DTI abnormalities in several white matter tracts such as posterior thalamic radiation pathways or superior regions of the thalamocortical and corticomotor tracts correlated with motor function measured by, e.g., Gross Motor Function Classification System (GMFCS) level (*n* = 28–34) (34, 41, 47). A review paper summarized the results of 22 DTI studies in CP and reported common findings of decrease FA (or increased MD) in the corticomotor and sensorimotor pathways, which correlated with clinical measures (48). Some research findings suggested that the CP injury in the somatosensory circuits might be more severe than that in the motor circuits, which may contribute more to motor impairment in CP (49). Further research is needed to unfold the mechanisms underlie sensorimotor impairment in CP and to improve detection of CP through neuroimaging.

Apart from neuroimaging, the abnormalities of CP have been identified *via* multivariate analysis using patients’ clinical data (perinatal history, CP type, CP frequency, motor function, degree of disability, etc.) and data from other sources such as electromyography (EMG) and bone mineral density (BMD) (32, 35–40, 42). For example, multivariate analyses have indicated that 85% of CP patients have oropharyngeal dysphagia (36), 13% have sleep disturbance (37), 70% have abnormal optic nerve head (40), and 50% have low BMD (42). Factors associated with these CP abnormalities have also been identified (35, 37, 39).

In addition, since neonatal encephalopathy can cause CP, detection of neonatal encephalopathy helps detect potential CP. Structural brain connectivity networks of infants with neonatal encephalopathy have been examined using diffusion tractography extracted from DTI images, and ML methods such as SVM have been applied to structural connectivity features to detect neonatal encephalopathy (50). Moreover, since epilepsy and seizure disorders are common in children with CP, electroencephalography (EEG) is used to detect co-occurring seizures in high-risk infants or children. ML approaches such as SVM and ANN have been applied to EEG features to identify ictal and interictal spikes and achieved high detection rate for seizures (51). Further, multivariate analysis has found that children with CP after perinatal or neonatal stroke are more likely to have severe disability, cognitive impairment or epilepsy than CP children after delayed stroke (32).

Taken together, multivariate analytic studies in CP detection have identified imaging markers such as intraventricular hemorrhage and PVL on cranial ultrasound (24), injury to white matter near the paracentral lobule or to the STN on T2 MRI images (33), and injury in the CSTs and the posterior thalamic radiation pathways on DTI images (34, 41, 46, 47), which are useful in detecting CP and differentiating CP subtypes. Multivariate analyses have also identified CP abnormalities and their associated factors from non-imaging data (32, 35–40, 42). Further research is needed to identify biomarkers at the early stages of the disease to improve the diagnosis of CP, reduce diagnosis delay, and allow early identification and intervention for CP.

## ML in Movement Assessment for CP Prediction

Studies reported applications of ML in movement assessment for CP prediction are summarized in Table 2 (52–61). Early identification of motor impairments in high-risk infants enables early detection of CP. The two atypical movement features (related to the cramped-synchronized general movements and the absence of fidgety movements) in general movement assessment are strong predictors for CP diagnosis (8–10). Based on these key motor impairment features, their movement characteristics and associated movement variables have been identified to detect movement impairments in high-risk infants (53–55, 57–59). ML approaches have made it possible to analyze recorded movement data and identify motor impairments automatically.

**Table 2:** Summary of studies with multivariate analytic and machine learning approaches in movement assessment and outcome evaluation in CP.

Study	Subject sample	Data	Methods	Main findings	Other findings
Meinecke et al. (52)	22 infants (7 at risk of CP, 15 healthy)	53 parameters extracted from recorded 3D movement data	Cluster analysis based on Euclidian distances and quadratic discriminant analysis used to find the best combined parameters and separate at risk infants from healthy ones	Overall detection rate (using an optimal combination of 8 parameters): 73% (sensitivity: 1.00; specificity: 0.70)	
Berge et al. (53)	14 infants with CP (who had four types of fidgety movements)	Motion features (1D, 2D, and Wigner-Ville time-frequency virtue/feature) extracted from video recordings of movements	Periodicity (fidgety movements characterized by periodic patterns); principal components analysis (PCA) for data reduction; Pattern recognition (compare movement patterns in video with known visual patterns of fidgety movements)	ENIGMA (a software tool) can assess general movements and detect fidgety movements in CP pts	
Adde et al. (54)	30 high-risk preterm and term infants (13 developed CP in 5 years vs. 17 non-CP)	Movement variables (e.g., quantity of motion, and centroid of motion to identify fidgety movements) extracted from video recordings	Mann–Whitney *U* test; Logistic regression to identify CP predictors; ROC analysis to assess CP classification accuracy	1/13 of pts had fidgety movements; predictor of CP: combined variable (centroid of motion STD, quantity of motion mean, quantity of motion STD); prediction accuracy of the combined variable: sensitivity: 85%; specificity: 88%	Combined variable had the highest prediction accuracy; ambulatory and non-ambulatory function was predicted correctly in 90% pts with CP
Heinze et al. (55)	4 infants with CP, vs. 19 healthy infants	32 features (including velocity and acceleration) extracted from measurement of accelerometers	Optimal parameter combinations selected by genetic algorithm; a decision tree-based classifier used to differentiate between pts’ and controls’ data	Overall detection rate: 88–92% for all measurements	The low-cost movement disorder detection system based on accelerometers is applicable to CP diagnosis in newborns
Alaqtash et al. (56)	4 pts with spastic diplegic CP, vs. 4 pts with multiple sclerosis, vs. 12 healthy controls	Gait features extracted from 3D ground reaction force data	NNC and ANN used to classify gait features into three groups; leave-one-out resampling	Classification accuracy (weighted average): 85% (using a combination of gait features); 95% (using an optimal set of six features)	
Karch et al. (57)	10 infants with spastic CP, vs. 53 non-CP infants	Stereotypy score of limb movements extracted from electromagnetic movement tracking recordings	A multi-segmental chain model used to calculate the joint centers and joint axes; dynamic time warping used to compute stereotype scores; ROC analysis used to assess CP classification accuracy	CP classification accuracy using stereotype score of upper lime movement: sensitivity: 90%; specificity: 96%	Using stereotype score of leg movement could not distinguish pts from controls
Stahl et al. (58)	82 infants (15 with CP, 67 healthy)	Motion features (such as motion distance and relative frequency) extracted from video movement recordings	Motion features selected to identify fidgety movements; SVM used to classify pts from controls; 10-fold cross-validation for classifier validation	Classification accuracy: with features of relative frequency: 93.7 ± 2.1%; sensitivity: 85.3 ± 2.8%; specificity: 95.5 ± 2.5%	Classification with other features (absolute motion distance and wavelet coefficient) had lower accuracy
Kanemaru et al. (59)	145 preterm infants (16 developed CP by 3 years of age, vs. 129 normal)	6 movement indices (average velocity of limb movement, number of movement units, kurtosis of acceleration, jerk index, etc.) extracted from video recordings	Fisher’s exact test and Mann–Whitney *U*-test to distinguish pts from controls	CP pts had higher jerk index in the legs (*p* < 0.01), average velocity of the arms (*p* < 0.05), and number of movement units of the arms (*p* < 0.05) than controls	Jerkiness of spontaneous movements in preterm infants at term age is useful for predicting CP
Wahid et al. (60)	51 children with diplegic CP vs. 34 healthy controls	Spatiotemporal gait data (physical properties, walking speed, etc.)	Multiple regression normalization and standard dimensionless equations used for data normalization; multiple regression normalization to identify the effects of AFO on gait in pts	Multiple regression normalization revealed difference in more spatiotemporal parameters in pts who walked with and without an AFO; after multiple regression normalization, most spatiotemporal parameters in pts with AFO became closer to those of controls	Multiple regression normalization may be useful in evaluating CP gait and gait classification
Parmar and Morris (61)	5 healthy subjects (who did exercises correctly, and also mimic the errors/mistakes in exercise made by CP pts)	Features (joint positions, angles) in the time domain (also transformed to the frequency domain) extracted from 78 training samples and 47 testing samples of physical exercises video recording	4 classifiers (SVM, NN, AdaBoosted decision tree, and DTW) used to distinguish good and erroneous exercises (in five sample exercises such as Blast-Off exercise in CP physical therapy)	Classification accuracy: 94.68% for AdaBoosted tree on joint data (in time domain); 90.89% for SVM on joint data (in frequency domain); 90.65% for SVM on joint data (in time domain); 90.3% for AdaBoosted tree on angle data (in time domain)	Classification accuracy: 90.13% for single-layer NN on joint data (in time domain); 87.63% for SVM on angle data (in frequency domain); 74.03% for DTW on angle data (in time domain)
Hemming et al. (62)	4,007 children with CP	Data from five CP registers (birth characteristics, severity of CP, level of impairment, socioeconomic status, etc.)	Kaplan–Meier survival estimates performed; Multivariate proportional hazards model fitted for survival analysis	Death rate: ~8%; rate of children who survived to 20 years of age: 85–94%; predictors of CP survival: The number and severity of impairment	Birth weight and socioeconomic status might have impact on survival in certain register regions
Kim et al. (63)	174 children with spastic CP who underwent SDR	Clinical data (age at surgery, types of CP, history of prematurity, motor function, history of seizures, etc.)	Univariate and multivariate logistic regression used to identify factors associated with surgical outcome	6.3% pts had a poor outcome; predictor of outcome: type of CP (diplegia, quadriplegia)	Preoperative diagnosis was a strong predictor; intellectual delay was significant only in univariate analysis
Golan et al. (64)	98 pts with spastic CP who underwent SDR	Data from hospital charts and radiographic spinal studies (preoperative and postoperative)	Univariate and multivariate regression analyses used to identify risk factors for spinal deformity	Risk factors for spinal deformity: CP severity; ambulatory function; age at surgery; gender	Factors associated with a lower rate of hyperlordosis: younger age at surgery and male gender
Majnemer et al. (65)	95 children with CP	Data from Child Health Questionnaire and Pediatric QOL Inventory (by pts and parents), and measurements (impairments, activity limitations, etc.)	Multivariate analysis used to identify determinants of QOL	Indicators of physical well-being: motor and other activity limitations; predictors of social-emotional adaptation: family functioning, behavioral difficulties, and motivation	47% pts had mild motor impairment
White-Koning et al. (66)	500 children with CP (in 7 countries in Europe)	Data from the Kidscreen questionnaire (by pts and parents)	Multivariate analysis used to identify factors associated the differences in parents’ and pts’ reports	Factors associated with the differences in parents’ and pts’ reports: high levels of stress in parenting (negative influence), self-reported severe child pain	Pts’ self-reports higher than parents’ in 8 domains, lower in the finances domain, and similar in the emotions domain
Long et al. (67)	71 pts with CP vs. 77 non-CP; all subjects underwent orthopedic surgery	Demographic, surgical, and medical data (intraoperative opioid dosing, postoperative ICU admission, postoperative oxygen desaturation, etc.)	Multivariate regression analysis used to determine intraoperative opioid dosing associated outcomes and other variables	CP pts received less intraoperative opioid than non-CP pts; predictors of postoperative ICU admission and postoperative oxygen desaturation: intraoperative opioid dosing	CP associated with decreased opioid dosing
Smits et al. (68)	116 pts with CP	3-year longitudinal data (motor function, intellectual capacity, etc.)	Univariate and multivariate analyses to investigate associations between the course of capabilities (e.g., in mobility) and CP-, child-, and family characteristics	Predictors of self-care: a model including level of gross motor function and intellectual capacity; predictors of mobility: a model only including level of gross motor function; predictors of social function: a model including level of bimanual function and paternal educational level	Greater increase in capabilities for higher level of functioning, except for level of paternal education
Sponseller et al. (69)	204 pts with CP who underwent spinal fusion surgery (at 7 institutions)	Clinical data of patient, laboratory, and surgical characteristics	Univariate and multivariate regression analysis to identify factors associated with infection development	6.4% patients developed deep wound infection; factors associated with deep wound infection: presence of a gastrostomy/gastrojejunostomy tube	
He et al. (70)	61 pts with spastic CP	Serial R- and S-baclofen plasma concentrations	Mixed-effects population model and a 2-compartment model used for population pharmacokinetics analysis of oral baclofen; a final multivariable model used to describe oral baclofen profiles	Mean population estimate of apparent clearance/F: 0.273 L/h/kg with 33.4% IIV; apparent volume of distribution (Vss/F): 1.16 L/kg with 43.9% IIV; average baclofen terminal half-life: 4.5 h	Determinants of apparent clearance: body weight, a possible genetic factor, and age
Kato et al. (71)	31 pts with CP and cervical myelopathy; 30 with CSM, all pts underwent posterior decompression surgery	Measurements of pedicle and placement of pedicle screws from CT scans	Multivariate analysis used to evaluate factors associated with the breach of cervical pedicle screws	23% CP pts and 7% CSM pts had pedicle sclerosis; pedicle sclerosis associated with a higher risk of breach	
Kruijsen-Terpstra et al. (72)	92 pts (2 years old) with CP	Longitudinal data (type of CP, GMFCS level, intellectual capacity, whether epilepsy, etc.)	Multivariate analysis used to identify determinants of development of self-care and mobility activities	Determinants of development of self-care activities: GMFCS and intellectual capacity; determinant of development of mobility activities: GMFCS	Self-care and mobility activity changes were less favorable in pts with severe CP
Shore et al. (73)	320 children with CP who underwent VDRO for treatment of hip displacement	Clinical data (Age, sex, GMFCS, preoperative radiography, use of botulinum toxin, surgical performance, surgeon volume, etc.)	Univariate and multivariate (Cox regression) analyses used to determine effects of the data variables on surgical success; Kaplan–Meier survivorship curve generated	92% success rate for GMFCS levels I and II vs. 76% success rate for GMFCS level V; predictor of surgical success: soft-tissue release at VDRO	37% surgical failure; predictors of surgical revision: younger age at surgery, increased GMFCS level, and lower annual surgical hip volume
Mo et al. (74)	206 children with CP who underwent surgical scoliosis correction	Clinical data (age, motor deficits, seizure history, verbal communication, mental retardation, Hydrocephalus severity, etc.)	Univariate and multivariate logistic regression used to identify factors causing poor IONM signals	Predictors of poor IONM signals: PVL, hydrocephalus, encephalomalacia; predictors of no signals: moderate or marked hydrocephalus, encephalomalacia	Predictors of no motor signal: focal PVL, moderate or marked hydrocephalus, encephalomalacia; predictors of no sensory signal: moderate hydrocephalus
Grecco et al. (75)	56 children with spastic CP	Clinical and neurophysiologic data (age, gross motor function, laterality of motor impairment, injury location and MEP)	Univariate and multivariate logistic regression analyses used to identify predictors of tDCS responses	Predictors of good responses to tDCS (and gait training): MEP (for 6-min walk test and gait speed), and subcortical injury (for gait kinematics and gross motor function)	The interaction of MEP and brain injury location predicted the responsiveness of tDCS
Minhas et al. (76)	1,746 pts who underwent orthopedic procedure (345 pts underweight, 952 pts normal weight, 209 overweight, 240 obese)	Clinical data (whether seizure, whether asthma, whether use steroid, surgical procedure, etc.)	Multivariate logistic regressions performed to evaluate the effect of BMI on complications	Risk factors for total and medical complications in spine, hip, and lower extremity procedures: underweight class	Weight was not associated with complications in tendon procedures; overweight and obesity not associated with increased risk for complications
Galarraga et al. (77)	115 children with CP who underwent (hip, ankle, foot, etc.) surgery	Preoperative data (36 physical examination variables and gait kinematics) and surgery data	PCA data dimension reduction; multi-regression analysis used to predict postoperative lower limb kinematics	Based on the kinematic angle, mean prediction errors on test vary from 4° (pelvic obliquity and hip adduction) to 10° (hip rotation and foot progression)	Mean prediction errors are smaller than the variability of gait parameters
Mann et al. (78)	128 pts with CP	Physical activity, physical, psychosocial and total QOL reported by parents, walking performance measured by a StepWatch device	Multivariate regression used to examine the relationship of physical activity and walking performance to QOL	Physical activity positively associated with physical and total QOL; walking performance positively associated with physical QOL	Participation level positively associated with psychosocial QOL

*AFO, ankle foot orthosis; AHA, assisting hand assessment; ANN, artificial neural networks; BMI, body mass index; CP, cerebral palsy; CSM, cervical spondylotic myelopathy; DTW, dynamic time warping; EMG, electromyographic; ENIGMA, enhanced interactive general movement assessment; GMFCS, Gross Motor Function Classification System; ICU, intensive care unit; IIV, interindividual variability; IONM, intraoperative neuromonitoring; MEP, motor-evoked potential; NN, neural networks; NNC, nearest neighbor classifier; Pts, patients; PVL, periventricular leukomalacia; QOL, quality of life; ROC, receiver operating characteristics; SDR, selective dorsal rhizotomy; surgeon volume, the number of procedures performed; SVM, support vector machines; tDCS, transcranial direct current stimulation; VDRO, proximal femoral varus derotation osteotomy*.

As a pioneer study, Meinecke et al. analyzed the 3D movement data of infants (*n* = 22, seven with CP) from video recordings, extracted an optimal combination of movement features with cluster analysis, and identified CP motor impairments with quadratic discriminant analysis (overall detection rate: 73%) (52). Further, the characteristics of fidgety movements and associated movement measurements have been identified to distinguish infants with movement impairments from those without (53, 54, 58). For example, extracting motion features related to fidgety movements (such as motion distance and relative frequency) from video recordings with an optical flow-based method, Stahl et al. examined the motion patterns of 82 infants (15 with CP), applied SVM classifier to detection of CP movement impairments, and achieved a good classification accuracy (93.7 ± 2.1% with features of relative frequency; sensitivity: 85.3 ± 2.8%; specificity: 95.5 ± 2.5%) (58).

Apart from video recordings, other movement recording systems such as accelerometers and electromagnetic movement tracking system have been employed for movement assessment and CP prediction. Heinze et al. examined the general movements of a group of newborns and infants (*n* = 23, 4 with CP) with accelerometers, selected optimal (combined) movement features with genetic algorithm, classified CP motor impairments with a decision tree-based classifier, and obtained overall detection rates of 88–92% (55). To distinguish the gait patterns between patients with CP (*n* = 4), patients with multiple sclerosis (*n* = 4), and healthy controls (*n* = 12), Alaqtash et al. extracted gait features from 3D ground reaction force data, compared the gait patterns of the three groups, and applied nearest-neighbor classifier and ANN to gait feature classification, which led to overall classification accuracies of 85% (ANN, with a combination of gait features) and 95% (after optimizing the gait features to an optimal set of six gait features) (56). Moreover, using electromagnetic movement tracking recordings, Karch et al. studied the general movements from 63 infants (10 with CP), extracted movement features such as joint centers, and computed stereotype scores with dynamic time warping, yielding a high CP classification accuracy with stereotype score of upper lime movement (sensitivity: 90%; specificity: 96%) (57). For a review on movement recognition techniques in general movement assessment for CP prediction in high-risk infants, see Ref. (12).

In addition, multivariate and ML approaches have been used in the assessment of physical therapy, and the effect of orthotic devices such as ankle foot orthosis on CP patients (55, 56). To evaluate the quality of exercises in CP physical therapy, Parmar and Morris (*n* = 5) applied four classifiers (SVM, neural networks, AdaBoosted decision tree, and dynamic time warping) to movement feature (joint and angle data in time or frequency domain) classification to identify correct or wrong exercise, and found that among the four classifiers, AdaBoosted decision tree performed the best with high classification accuracies (94.68% for joint data; 90.3% for angle data) (56).

However, these machine-learning-based movement assessment studies are at the early stage of research. For example, the subjects in the study of Alaqtash et al. (56) were healthy subjects (*n* = 5), the movement data of wrong exercises (with errors) were simulated data, thus, the results were preliminary. Further research is needed to apply machine-learning methods to real movement data of patients with CP. In addition, the sample sizes of patients with CP in these machine-learning-based movement assessment studies are small (*n* = 4–15), studies with large samples are needed to further verify and improve these machine-learning methods. Further, the performance of these machine-learning studies or classification systems may be improved by optimizing the data processing chains (feature extraction, feature selection, classification, and verification). For details on optimizing data processing chains to improve classification performance, see Ref. (79).

Recently, Marschik et al. proposed an integrated system called a fingerprint model that monitors the movement and speech-language development of new-born babies and infants at risk and automatically detects neurodevelopmental disorders such as CP by multidimensional data analysis and machine-learning approaches (13). Although it is challenging (in technical details), the fingerprint model enables neurological assessment of at-risk infants in an objective and quantitative manner and facilitates early detection of CP and other neurodevelopmental disorders, which may be the future direction of pediatric clinical practice.

## Multivariate Analysis in CP Outcome Evaluation

Although there is no cure for CP, currently, treatment effects and outcomes in CP patients have been studied extensively. Multivariate approaches have been applied to outcome assessment (including survival analysis) in CP, and Table 2 (the latter part) provides a summary of these studies (62–78). The majority of the outcome studies employed a two-step approach: first, univariate analysis is used to identify variables that are associated with outcome; second, multivariate analysis is used to further examine the variables indicated by the univariate analysis and identify outcome predictors. A large sample of children with CP (*n* = 4,007) in UK were studied, and multivariate survival analysis indicated that the death rate was ~8, 85–94% of the children survived to age 20 years old, and the best predictors of CP survival were the number and severity of impairments (62). The multivariate outcome studies in CP fall into three categories: (1) outcome evaluation of medication and supportive treatments; (2) surgical outcome evaluation; and (3) quality of life (QOL) evaluation.

### Outcome Evaluation of Medication and Supportive Treatments

The effect of commonly used medication oral baclofen on children with CP has been assessed with a multivariate model for the population pharmacokinetics analysis (*n* = 61), and it has been found that baclofen dosage based on body weight was appropriate to treat patients (≥2 years old), and determinants of apparent clearance in these children included body weight, a possible genetic factor, and age (70). Plasticity (shown as increased FA in the CSTs on DTI and improved motor function measures) induced by combined therapy (botulinum followed by physiotherapy) in children with spastic quadriplegia (*n* = 8) has been reported (80), while a later DTI study indicated that the addition of botulinum to physiotherapy did not influence the outcome at 6 months in children with spastic diplegic CP (*n* = 18) (81). DTI has also been used to evaluate the motor function outcomes of hemiplegic CP patients after rehabilitation treatment and DTI measurements such as the fiber number and FA of bilateral CSTs were correlated with functional level of hemiplegia scale (82). The quality of exercises in CP physical therapy has been evaluated with several classifiers, and AdaBoosted decision tree obtained good detection rate of exercise errors (61). In addition, multivariate outcome evaluation of therapies such as transcranial direct current stimulation (tDCS) has been performed (75). Grecco et al. investigated the functional outcome of tDCS in children with CP (*n* = 56), and multivariate logistic regression analyses identified that the presence of motor evoked potential was a predictor for walk test and gait speed, subcortical injury was a predictor for gross motor function, and both of them were predictors of motor function gain arise from tDCS combined with gait training in these patients (75). However, there are few outcome studies of medications and supportive treatments in CP using multivariate analyses, and multivariate analysis may play a bigger role in such outcome evaluation to reveal the true therapeutic effects of these treatments and their outcomes in CP patients.

### Surgical Outcome Evaluation

A number of multivariate studies have investigated the outcomes of surgical procedures in CP (63, 64, 67, 71, 74–77). For example, the outcomes of SDR surgery have been studied in children with CP and outcome-associated factors have been identified (63, 64). Kim et al. examined factors associated with poor outcome of SDR surgery in pediatric patients with CP (*n* = 174) using multivariate logistic regression, and found that the poor-outcome rate was 6.3%, and the type of CP disability (diplegia, quadriplegia, etc.) was the predictor of poor outcome after SDR surgery (63). Further, Golan et al. evaluated the risk of spinal deformity in children with CP after SDR surgery (*n* = 98) and multivariate regression analysis identified several risk factors for spinal deformity: CP severity, ambulatory function, age at surgery, and gender (64). In addition, Long et al. investigated intraoperative opioid dosing and associated outcomes with multivariate regression in children with CP (*n* = 71) who underwent orthopedic surgery and reported that less intraoperative opioid was administered to CP children than non-CP children, and intraoperative opioid dosing was the outcome predictor of postoperative oxygen desaturation and ICU admission (67). Shore et al. examined the surgical outcome of children with CP (*n* = 320) who underwent proximal femoral varus derotation osteotomy (VDRO) for treatment of hip displacement, and found that success rate was 92% for GMFCS levels I and II vs. 76% for GMFCS level V, and multivariate analysis indicated that soft-tissue release during the VDRO procedure was the predictor of surgical success (73). The surgical outcome predictors identified by these studies are useful for outcome prediction for individual CP patients.

Complications after CP surgical treatments have also been investigated. Using multivariate regression analysis, Sponseller et al. studied deep wound infection after spinal fusion surgery at seven institutions in pediatric patients with CP (*n* = 204) and found that 6.4% of patients developed deep wound infection following surgery, and presence of a gastrostomy/gastrojejunostomy tube was the factor associated with infection (69). In addition, Kato et al. investigated cervical spine in patients with athetoid CP who underwent posterior decompression surgery (*n* = 31) and multivariate analysis showed that pedicle sclerosis associated with a higher risk of breach of cervical pedicle screws (71). Further, Minhas et al. evaluated the effect of body mass index class on complications after orthopedic surgery in children with CP (*n* = 1,746) and multivariate logistic regression analysis revealed that underweight status was the risk factor for complications in osteotomies and spine surgery (76). The risk factors identified by these studies are helpful to avoid the surgical complications and improve surgical treatments in CP.

In children with CP who underwent surgery, intraoperative neuromonitoring (IONM) often fail (failure rate 61%) (74). Mo et al. studied IONM in children with CP who underwent surgical scoliosis correction (*n* = 206) and multivariate logistic regression analysis revealed that PVL, hydrocephalus, and encephalomalacia were the predictors of poor IONM signals, while moderate or marked hydrocephalus and encephalomalacia were the predictors of no signals (74). Further, outcome prediction of CP surgical procedures has been explored in a recent study. Galarraga et al. examined children with CP who underwent (hip, ankle, foot, etc.) surgery (*n* = 115), and multi-regression analysis revealed that preoperative and surgical data could predict postoperative kinematics, and mean prediction errors (varying from 4° to 10°) were smaller compared with the variability of gait parameters (77). These results are encouraging because they indicated that the postsurgical kinematics of patients with CP could be predicted (relatively accurately with small mean prediction errors) using presurgical and surgical data, which allows an estimate of postsurgical outcome ahead of time.

### QOL Evaluation

Quality of life in physical ability, intellectual ability, self-care, and other aspects of life is an important outcome in CP. Multivariate analysis has been frequently used to assess QOL in patients with CP (65, 66, 68, 72, 78), and factors associated with physical QOL and self-care have been identified. For example, a multivariate analysis on QOL data of infants with CP (*n* = 92) identified GMFCS and intellectual capacity as the associated factors of self-care activity development, and GMFCS as the associated factors of mobility activities development (72). Further, a recent multivariate analysis showed that physical activity was positively associated with physical and total QOL in patients with CP (*n* = 128), and walking performance was positively associated with physical QOL (78). The factors identified by these studies may improve the QOL of patients with CP.

Taken together, since there is no cure for CP yet, and the death rate of CP is high (~8%), there is much to do to improve the outcomes of CP, and multivariate analytic approaches may play a bigger role in meeting such clinical demands. Surgical outcome predictors and risk factors for complications in CP surgical treatments have been identified by a number of multivariate outcome studies (63, 64, 67, 69, 71, 73, 76), which are useful not only for outcome evaluation and prediction but also for avoiding complications and improving surgical treatments in CP. However, there are few outcome studies for medications and supportive treatments (such as physical therapy) in CP using multivariate analysis. Thus, further research is needed to evaluate the outcomes of medications and supportive treatments, and multivariate analysis may play a bigger role in such outcome evaluation to reveal the true therapeutic effects of these treatments and their outcomes in CP patients, and help improve the outcomes of these treatments for patients with CP.

## Summary

Multivariate analysis has been applied to several areas in CP research such as identification of risk factors for CP, detection of CP and identification of CP abnormalities, movement assessment for CP prediction, and outcome assessment. The studies reviewed in this paper have demonstrated that multivariate analytic and ML approaches have made it possible to analyze movement recordings and identify CP movement impairments automatically. In addition, outcome predictors for surgical treatments have been identified by multivariate outcome studies. To make the multivariate analytic and ML approaches useful in clinical settings, further research with large samples is needed to verify and improve these methods in CP detection, movement assessment, and outcome evaluation/prediction. As multivariate analysis, ML and data processing technologies advance in the era of Big Data, it is expected that multivariate analysis and ML will play a bigger role in improving the diagnosis and treatment of CP to reduce mortality and morbidity rates, and enhance patient care for children with CP.

## Author Contributions

JZ reviewed the multivariate analytic studies in cerebral palsy and wrote up the manuscript.

## Conflict of Interest Statement

The author declares that the research was conducted in the absence of any commercial or financial relationships that could be construed as a potential conflict of interest.

## References

[1] Herskind A Greisen G Nielsen JB Early identification and intervention in cerebral palsy. Dev Med Child Neurol (2015) 57(1):29–36. 10.1111/dmcn.12531 25041565

[2] McIntyre S Morgan C Walker K Novak I Cerebral palsy – don’t delay. Dev Disabil Res Rev (2011) 17:114–29. 10.1002/ddrr.1106 23362031

[3] Novak I Hines M Goldsmith S Barclay R Clinical prognostic messages from a systematic review about cerebral palsy. Pediatrics (2012) 130:e1285–312. 10.1542/peds.2012-0924 23045562

[4] Reid S Carlin J Reddihough D. Distribution of motor types in cerebral palsy: how do registry data compare? Dev Med Child Neurol (2011) 53:233–8. 10.1111/j.1469-8749.2010.03844.x 21166669

[5] NIH (National Institute of Neurological Disorders and Stroke). Cerebral Palsy: Hope through Research. (2017). Available from: https://www.ninds.nih.gov/Disorders/Patient-Caregiver-Education/Hope-Through-Research/Cerebral-Palsy-Hope-Through-Research#3104_2

[6] Krageloh-Mann I Horber V. The role of magnetic resonance imaging in elucidating the pathogenesis of cerebral palsy: a systematic review. Dev Med Child Neurol (2007) 49:144–51. 10.1111/j.1469-8749.2007.00144.x 17254004

[7] Prechtl HFR Qualitative changes of spontaneous movements in fetus and preterm infant are a marker of neurological dysfunction. Early Hum Dev (1990) 23(3):151–8. 10.1016/0378-3782(90)90011-7 2253578

[8] Einspieler C Prechtl HFR. Prechtl’s assessment of general movements: a diagnostic tool for the functional assessment of the young nervous system. Ment Retard Dev Disabil Res Rev (2005) 11:61–7. 10.1002/mrdd.20051 15856440

[9] Einspieler C Marschik PB Bos AF Ferrari F Cioni G Prechtl HFR Early markers for cerebral palsy: insights from the assessment of general movements. Future Neurol (2012) 7(6):709–17. 10.2217/fnl.12.60

[10] Spittle AJ Orton J Cerebral palsy and developmental coordination disorder in children born preterm. Semin Fetal Neonatal Med (2013) 19(2):84–9. 10.1016/j.siny.2013.11.005 24290908

[11] Bosanquet M Copeland L Ware R Boyd R. A systematic review of tests to predict cerebral palsy in young children. Dev Med Child Neurol (2013) 55(5):418–26. 10.1111/dmcn.12140 23574478

[12] Marcroft C Khan A Embleton ND Trenell M Plötz T. Movement recognition technology as a method of assessing spontaneous general movements in high risk infants. Front Neurol (2015) 5:284. 10.3389/fneur.2014.00284 25620954 PMC4288331

[13] Marschik PB Pokorny FB Peharz R Zhang D O’Muircheartaigh J Roeyers H A novel way to measure and predict development: a heuristic approach to facilitate the early detection of neurodevelopmental disorders. Curr Neurol Neurosci Rep (2017) 17(5):43. 10.1007/s11910-017-0748-8 28390033 PMC5384955

[14] Levman J Takahashi E. Multivariate analyses applied to fetal, neonatal and pediatric MRI of neurodevelopmental disorders. Neuroimage Clin (2015) 9:532–44. 10.1016/j.nicl.2015.09.017 26640765 PMC4625213

[15] Levman J Takahashi E. Pre-adult MRI of brain cancer and neurological injury: multivariate analyses. Front Pediatr (2016) 4:65. 10.3389/fped.2016.00065 27446888 PMC4917540

[16] McLachlan GJ Discriminant Analysis and Statistical Pattern Recognition. Hoboken, NJ: Wiley Interscience (2004).

[17] Vapnik VN The Nature of Statistical Learning Theory. New York: Springer (1995).

[18] Yegnanarayana B Artificial Neural Networks. New Delhi: PHI Learning Pvt. Ltd (2009).

[19] Breiman L Random forests. Mach Learn (2001) 45:5–32. 10.1023/A:1010933404324

[20] Manton KG Lowrimore G Yashin A Kovtun M Cluster Analysis: Overview. Hoboken, NJ: Wiley StatsRef: Statistics Reference Online (2014).

[21] Cummins SK Nelson KB Grether JK Velie EM. Cerebral palsy in four northern California counties, births 1983 through 1985. J Pediatr (1993) 123(2):230–7. 10.1016/S0022-3476(05)81693-2 8345418

[22] Pinto-Martin JA Riolo S Cnaan A Holzman C Susser MW Paneth N Cranial ultrasound prediction of disabling and nondisabling cerebral palsy at age two in a low birth weight population. Pediatrics (1995) 95(2):249–54. 7838643

[23] Bax M Tydeman C Flodmark O. Clinical and MRI correlates of cerebral palsy: the European Cerebral Palsy Study. JAMA (2006) 296(13):1602–8. 10.1001/jama.296.13.1602 17018805

[24] Allan WC Vohr B Makuch RW Katz KH Ment LR Antecedents of cerebral palsy in a multicenter trial of indomethacin for intraventricular hemorrhage. Arch Pediatr Adolesc Med (1997) 151(6):580–5. 10.1001/archpedi.1997.02170430046010 9193243

[25] Kim JN Namgung R Chang W Oh CH Shin JC Park ES Prospective evaluation of perinatal risk factors for cerebral palsy and delayed development in high risk infants. Yonsei Med J (1999) 40(4):363–70. 10.3349/ymj.1999.40.4.363 10487140

[26] Golomb MR Garg BP Saha C Azzouz F Williams LS. Cerebral palsy after perinatal arterial ischemic stroke. J Child Neurol (2008) 23(3):279–86. 10.1177/0883073807309246 18305317

[27] Han TR Bang MS Lim JY Yoon BH Kim IW. Risk factors of cerebral palsy in preterm infants. Am J Phys Med Rehabil (2002) 81(4):297–303. 10.1097/00002060-200204000-00011 11953548

[28] Zhong Y Wu J Wu K Wen R Hou G Peng D Evaluation of risk factors associated with cerebral palsy in children of Leshan Prefecture, Sichuan: a case control study on 308 cases. Zhonghua Yu Fang Yi Xue Za Zhi (2002) 36(5):323–6. 12411193

[29] Miamoto CB Pereira LJ Paiva SM Pordeus IA Ramos-Jorge ML Marques LS. Prevalence and risk indicators of temporomandibular disorder signs and symptoms in a pediatric population with spastic cerebral palsy. J Clin Pediatr Dent (2011) 35(3):259–63. 10.17796/jcpd.35.3.738t75v74l1m1p22 21678667

[30] Abdullahi H Satti M Rayis DA Imam AM Adam I Intra-partum fever and cerebral palsy in Khartoum, Sudan. BMC Res Notes (2013) 24(6):163 10.1186/1756-0500-6-163 PMC3641995 23618409

[31] Yu T Rong L Wang Q You Y Fu JX Kang LM Influence of neonatal diseases and treatments on the development of cerebral palsy in preterm infant. Sichuan Da Xue Xue Bao Yi Xue Ban (2013) 44(2):270–3. 23745270

[32] Golomb MR Saha C Garg BP Azzouz F Williams LS. Association of cerebral palsy with other disabilities in children with perinatal arterial ischemic stroke. Pediatr Neurol (2007) 37(4):245–9. 10.1016/j.pediatrneurol.2007.06.003 17903667 PMC2587173

[33] Griffiths PD Radon MR Crossman AR Zurakowski D Connolly DJ Anatomic localization of dyskinesia in children with “profound” perinatal hypoxic-ischemic injury. AJNR Am J Neuroradiol (2010) 31(3):436–41. 10.3174/ajnr.A1854 19875467 PMC7963969

[34] Yoshida S Hayakawa K Yamamoto A Okano S Kanda T Yamori Y Quantitative diffusion tensor tractography of the motor and sensory tract in children with cerebral palsy. Dev Med Child Neurol (2010) 52(10):935–40. 10.1111/j.1469-8749.2010.03669.x 20412261

[35] Coppola G Fortunato D Mainolfi C Porcaro F Roccaro D Signoriello G Bone mineral density in a population of children and adolescents with cerebral palsy and mental retardation with or without epilepsy. Epilepsia (2012) 53(12):2172–7. 10.1111/j.1528-1167.2012.03639.x 22958083

[36] Benfer KA Weir KA Bell KL Ware RS Davies PS Boyd RN. Oropharyngeal dysphagia and gross motor skills in children with cerebral palsy. Pediatrics (2013) 131(5):e1553–62. 10.1542/peds.2012-3093 23589816

[37] Romeo DM Brogna C Musto E Baranello G Pagliano E Casalino T Sleep disturbances in preschool age children with cerebral palsy: a questionnaire study. Sleep Med (2014) 15(9):1089–93. 10.1016/j.sleep.2014.05.008 25091533

[38] Adler C Berweck S Lidzba K Becher T Staudt M. Mirror movements in unilateral spastic cerebral palsy: specific negative impact on bimanual activities of daily living. Eur J Paediatr Neurol (2015) 19(5):504–9. 10.1016/j.ejpn.2015.03.007 26004994

[39] Tao W Zhang X Chen X Wu D Zhou P Multi-scale complexity analysis of muscle coactivation during gait in children with cerebral palsy. Front Hum Neurosci (2015) 9:367 10.3389/fnhum.2015.00367 26257622 PMC4510417

[40] Ghate D Vedanarayanan V Kamour A Corbett JJ Kedar S. Optic nerve morphology as marker for disease severity in cerebral palsy of perinatal origin. J Neurol Sci (2016) 368:25–31. 10.1016/j.jns.2016.06.029 27538596

[41] Reid LB Cunnington R Boyd RN Rose SE. Surface-based fMRI-driven diffusion tractography in the presence of significant brain pathology: a study linking structure and function in cerebral palsy. PLoS One (2016) 11(8):e0159540. 10.1371/journal.pone.0159540 27487011 PMC4972431

[42] Tosun A Erisen Karaca S Unuvar T Yurekli Y Yenisey C Omurlu IK. Bone mineral density and vitamin D status in children with epilepsy, cerebral palsy, and cerebral palsy with epilepsy. Childs Nerv Syst (2017) 33(1):153–8. 10.1007/s00381-016-3258-0 27757568

[43] Kayem G Mandelbrot L Haddad B Use of magnesium sulfate in obstetrics. Gynecol Obstet Fertil (2012) 40(10):605–13. 10.1016/j.gyobfe.2012.08.005 22995056

[44] Crowther CA Middleton PF Voysey M Askie L Duley L Pryde PG Assessing the neuroprotective benefits for babies of antenatal magnesium sulphate: an individual participant data meta-analysis. PLoS Med (2017) 14(10):e1002398. 10.1371/journal.pmed.1002398 28976987 PMC5627896

[45] Jacobs SE Tarnow-Mordi WO Therapeutic hypothermia for newborn infants with hypoxic–ischaemic encephalopathy. J Paediatr Child H (2010) 46:568–76. 10.1111/j.1440-1754.2010.01880.x 20846275

[46] Thomas B Eyssen M Peeters R Molenaers G Van Hecke P De Cock P Quantitative diffusion tensor imaging in cerebral palsy due to periventricular white matter injury. Brain (2005) 128(Pt 11):2562–77. 10.1093/brain/awh600 16049045

[47] Hoon AH Jr Stashinko EE Nagae LM Lin DD Keller J Bastian A Sensory and motor deficits in children with cerebral palsy born preterm correlate with diffusion tensor imaging abnormalities in thalamocortical pathways. Dev Med Child Neurol (2009) 51(9):697–704. 10.1111/j.1469-8749.2009.03306.x 19416315 PMC2908264

[48] Scheck SM Boyd RN Rose SE. New insights into the pathology of white matter tracts in cerebral palsy from diffusion magnetic resonance imaging: a systematic review. Dev Med Child Neurol (2012) 54(8):684–96. 10.1111/j.1469-8749.2012.04332.x 22646844

[49] Wilke M Staudt M Does damage to somatosensory circuits underlie motor impairment in cerebral palsy? Dev Med Child Neurol (2009) 51:686–7. 10.1111/j.1469-8749.2009.03332.x 19709138

[50] Ziv E Tymofiyeva O Ferriero DM Barkovich AJ Hess CP Xu D. A machine learning approach to automated structural network analysis: application to neonatal encephalopathy. PLoS One (2013) 8(11):e78824. 10.1371/journal.pone.0078824 24282501 PMC3840059

[51] Cabrerizo M Ayala M Goryawala M Jayakar P Adjouadi M. A new parametric feature descriptor for the classification of epileptic and control EEG records in pediatric population. Int J Neural Syst (2012) 22:1250001. 10.1142/S0129065712500013 23627587

[52] Meinecke L Breitbach-Faller N Bartz C Damen R Rau G Disselhorst-Klug C. Movement analysis in the early detection of newborns at risk for developing spasticity due to infantile cerebral palsy. Hum Mov Sci (2006) 25(2):125–44. 10.1016/j.humov.2005.09.012 16458381

[53] Berge PR Adde L Espinosa G Stavdahl Ø ENIGMA – enhanced interactive general movement assessment. Expert Syst Appl (2008) 34(4):2664–72. 10.1016/j.eswa.2007.05.024

[54] Adde L Helbostad JL Jensenius AR Taraldsen G Grunewaldt KH Støen R. Early prediction of cerebral palsy by computer-based video analysis of general movements: a feasibility study. Dev Med Child Neurol (2010) 52(8):773–8. 10.1111/j.1469-8749.2010.03629.x 20187882

[55] Heinze F Hesels K Breitbach-Faller N Schmitz-Rode T Disselhorst-Klug C. Movement analysis by accelerometry of newborns and infants for the early detection of movement disorders due to infantile cerebral palsy. Med Biol Eng Comput (2010) 48(8):765–72. 10.1007/s11517-010-0624-z 20446047

[56] Alaqtash M Sarkodie-Gyan T Yu H Fuentes O Brower R Abdelgawad A Automatic classification of pathological gait patterns using ground reaction forces and machine learning algorithms. Conf Proc IEEE Eng Med Biol Soc (2011):453–7. 10.1109/IEMBS.2011.6090063 22254346

[57] Karch D Kang K-S Wochner K Philippi H Hadders-Algra M Pietz J Kinematic assessment of stereotypy in spontaneous movements in infants. Gait Posture (2012) 36(2):307–11. 10.1016/j.gaitpost.2012.03.017 22503388

[58] Stahl A Schellewald C Stavdahl O Aamo OM Adde L Kirkerod H. An optical flow-based method to predict infantile cerebral palsy. IEEE Trans Neural Syst Rehabil Eng (2012) 20(4):605–14. 10.1109/TNSRE.2012.2195030 22531824

[59] Kanemaru N Watanabe H Kihara H Nakano H Nakamura T Nakano J Jerky spontaneous movements at term age in preterm infants who later developed cerebral palsy. Early Hum Dev (2014) 90(8):387–92. 10.1016/j.earlhumdev.2014.05.004 24951073

[60] Wahid F Begg R Sangeux M Halgamuge S Ackland DC The effects of an ankle foot orthosis on cerebral palsy gait: a multiple regression analysis. Conf Proc IEEE Eng Med Biol Soc (2015) 5509–12. 10.1109/EMBC.2015.7319639 26737539

[61] Parmar P Morris BT Measuring the quality of exercises. Conf Proc IEEE Eng Med Biol Soc (2016) 2241–4. 10.1109/EMBC.2016.7591175 28268775

[62] Hemming K Hutton JL Colver A Platt MJ. Regional variation in survival of people with cerebral palsy in the United Kingdom. Pediatrics (2005) 116(6):1383–90. 10.1542/peds.2005-0259 16322162

[63] Kim HS Steinbok P Wickenheiser D. Predictors of poor outcome after selective dorsal rhizotomy in treatment of spastic cerebral palsy. Childs Nerv Syst (2006) 22(1):60–6. 10.1007/s00381-005-1160-2 15906043

[64] Golan JD Hall JA O’Gorman G Poulin C Benaroch TE Cantin MA Spinal deformities following selective dorsal rhizotomy. J Neurosurg (2007) 106(6 Suppl):441–9. 10.3171/ped.2007.106.6.441 17566400

[65] Majnemer A Shevell M Rosenbaum P Law M Poulin C. Determinants of life quality in school-age children with cerebral palsy. J Pediatr (2007) 151(5):470–5. 10.1016/j.jpeds.2007.04.014 17961687

[66] White-Koning M Arnaud C Dickinson HO Thyen U Beckung E Fauconnier J Determinants of child-parent agreement in quality-of-life reports: a European study of children with cerebral palsy. Pediatrics (2007) 120(4):e804–14. 10.1542/peds.2006-3272 17908738

[67] Long LS Ved S Koh JL. Intraoperative opioid dosing in children with and without cerebral palsy. Paediatr Anaesth (2009) 19(5):513–20. 10.1111/j.1460-9592.2009.02980.x 19453584

[68] Smits DW Ketelaar M Gorter JW van Schie P Dallmeijer A Jongmans M Development of daily activities in school-age children with cerebral palsy. Res Dev Disabil (2011) 32(1):222–34. 10.1016/j.ridd.2010.09.025 21041062

[69] Sponseller PD Jain A Shah SA Samdani A Yaszay B Newton PO Deep wound infections after spinal fusion in children with cerebral palsy: a prospective cohort study. Spine (Phila Pa 1976) (2013) 38(23):2023–7. 10.1097/BRS.0b013e3182a83e59 23963019

[70] He Y Brunstrom-Hernandez JE Thio LL Lackey S Gaebler-Spira D Kuroda MM Population pharmacokinetics of oral baclofen in pediatric patients with cerebral palsy. J Pediatr (2014) 164(5):1181–8. 10.1016/j.jpeds.2014.01.029 24607242 PMC3992203

[71] Kato S Shoda N Chikuda H Seichi A Takeshita K Morphological characteristics of cervical spine in patients with athetoid cerebral palsy and the accuracy of pedicle screw placement. Spine (Phila Pa 1976) (2014) 39(8):E508–13. 10.1097/BRS.0000000000000234 24480949

[72] Kruijsen-Terpstra AJ Ketelaar M Verschuren O Smits DW Jongmans MJ Gorter JW. Determinants of developmental gain in daily activities in young children with cerebral palsy. Phys Occup Ther Pediatr (2015) 35(3):265–79. 10.3109/01942638.2014.957429 25232647

[73] Shore BJ Zurakowski D Dufreny C Powell D Matheney TH Snyder BD. Proximal femoral varus derotation osteotomy in children with cerebral palsy: the effect of age, gross motor function classification system level, and surgeon volume on surgical success. J Bone Joint Surg Am (2015) 97(24):2024–31. 10.2106/JBJS.O.00505 26677236

[74] Mo AZ Asemota AO Venkatesan A Ritzl EK Njoku DB Sponseller PD Why no signals? Cerebral anatomy predicts success of intraoperative neuromonitoring during correction of scoliosis secondary to cerebral Palsy. J Pediatr Orthop (2015) 37(8):e451–8. 10.1097/BPO.0000000000000707 26683503

[75] Grecco LA Oliveira CS Galli M Cosmo C Duarte Nde A Zanon N Spared primary motor cortex and the presence of MEP in cerebral palsy dictate the responsiveness to tDCS during gait training. Front Hum Neurosci (2016) 10:361. 10.3389/fnhum.2016.00361 27486393 PMC4949210

[76] Minhas SV Chow I Otsuka NY. The effect of body mass index on postoperative morbidity after orthopaedic surgery in children with cerebral palsy. J Pediatr Orthop (2016) 36(5):505–10. 10.1097/BPO.0000000000000475 25929775

[77] Galarraga COA Vigneron V Dorizzi B Khouri N Desailly E. Predicting postoperative gait in cerebral palsy. Gait Posture (2017) 52:45–51. 10.1016/j.gaitpost.2016.11.012 27871017

[78] Mann K Tsao E Bjornson KF Physical activity and walking performance: influence on quality of life in ambulatory children with cerebral palsy (CP). J Pediatr Rehabil Med (2016) 9(4):279–86. 10.3233/PRM-160395 27935563 PMC6171113

[79] Zhang J Multivariate analysis in pediatric brain tumor. Int J Radiol Radiat Therapy (2017) 2(6):00045 10.15406/ijrrt.2017.02.00045

[80] Chaturvedi SK Rai Y Chourasia A Goel P Paliwal VK Garg RK Comparative assessment of therapeutic response to physiotherapy with or without botulinum toxin injection using diffusion tensor tractography and clinical scores in term diplegic cerebral palsy children. Brain Dev (2013) 35(7):647–53. 10.1016/j.braindev.2012.10.012 23165172

[81] Trivedi R Gupta RK Shah V Tripathi M Rathore RK Kumar M Treatment-induced plasticity in cerebral palsy: a diffusion tensor imaging study. Pediatr Neurol (2008) 39(5):341–9. 10.1016/j.pediatrneurol.2008.07.012 18940558

[82] Kim JH Kwon YM Son SM. Motor function outcomes of pediatric patients with hemiplegic cerebral palsy after rehabilitation treatment: a diffusion tensor imaging study. Neural Regen Res (2015) 10(4):624–30. 10.4103/1673-5374.155438 26170825 PMC4424757

